# Do Cardiovascular Diseases Significantly Influence Healthy Aging?

**DOI:** 10.3390/ijerph18147226

**Published:** 2021-07-06

**Authors:** Simona-Andreea Apostu, Valentina Vasile, Valentin Sava

**Affiliations:** 1 Department of Statistics and Econometrics, Bucharest University of Economic Studies, 010552 Bucharest, Romania; 2 Institute of National Economy, 050771 Bucharest, Romania; valentinavasile2009@gmail.com; 3 Department of Economics, “Dunărea de Jos” University of Galati, 800201 Galați, Romania; valentin.sava15@gmail.com

**Keywords:** healthy aging, longevity, education, cardiovascular disease, GDP, income, regression analysis, principal component analysis, cluster analysis

## Abstract

Population development is reflected by sustainable development indicators, among them are the indicators describing longevity and healthy aging. Longevity is reflected by life expectancy, and healthy aging is reflected by healthy life expectancy; high values of these indicators reflect good conditions of living for people. Life expectancy and healthy life expectancy analyses are of big interest among academics, policymakers, medical researchers, and others in order to direct the flow of funds in the most effective way possible to the population groups in most need. High life expectancy and low birth rate will lead to aging of the population, having profound implications on the school age population, politics, healthcare, labor force, social protection, social security issues, and public finances. Healthy life expectancy reflects health conditions, including the impacts of mortality and morbidity. As cardiovascular disease causes more than half of all deaths across Europe, this paper examines the influence of cardiovascular disease on longevity and healthy aging across Europe. The methodology was chosen so as to test the research hypotheses: (a) principal component analysis provided the socio-economic factors that are correlated to longevity and healthy aging; (b) regression analysis identified the relationship between healthy aging and cardiovascular disease; and (c) hierarchical cluster analysis allowed us to find common features of the groups of countries according to healthy aging and longevity.

## 1. Introduction

Longevity through life expectancy reflects the average number of years a person has to live if they would live the rest of their life under the age-related mortality of the reference period (Eurostat). Each person’s life expectancy changes as the person ages or mortality trends change. Life expectancy at certain ages is very important in shaping life-cycle policies by gender, such as employment policies, retirement policies, and health care policies.

Healthy aging is defined by the World Health Organization (WHO) as “the process of developing and maintaining the functional ability that enables wellbeing in older age” and was the focus of WHO’s work on aging between 2015 and 2030 [1]. Healthy aging is reflected by life expectancy at 65 years of age, an important indicator for assessing population longevity by the aging phenomenon, as it is known that older people present poorer health and a higher mortality compared to younger people. Healthy life expectancy was introduced by the World Health Organization (WHO) as a summary measure of the health level attained by populations in the World Health Report 2000 [1].

In recent times, life expectancy has increased slowly and steadily [2] due to declining death rates caused by epidemics, pandemics, famine, and war infectious diseases [3,4] in countries with high variability [5,6].

Life expectancy is correlated with education; a high education level implies a low mortality rate and, consequently, a high life expectancy. Regarding education, people with a higher level of education live longer than others. For example, in Estonia, people with higher education live about 14 years longer than those with primary education, and, in Bulgaria and Slovakia, the difference in length of life in relation to education is 10 years. The smallest differences between life expectancy for persons with higher education and those with primary education in 2013 were Croatia—3.2 years, Italy—4.0 years, and Portugal—4.0 years [7].

The expectation of a healthy life at birth, which is the number of years expected for a person to live without health problems, was 65.1 years for women and 64.2 years for men in 2019 in the European Union. The number of years a person is expected to live without health problems was higher for women than for men in the 20 Member States, and, among them, there were four countries where this difference was greater than three years: Bulgaria, Estonia, Lithuania, and Poland.

The factors that contribute to better health and many years of life for people with higher education are environment, occupation, lifestyle, access to information, and quality healthcare.

Life expectancy and healthy life expectancy are valuable indicators for sustainable development, characterizing economic and social development. Life expectancy and healthy life expectancy have been the subject of many studies over time; in Europe, they have been registered as an ascending trend.

According to the “World Health Statistics 2016. Monitoring health for the SDGs” report, the average life expectancy calculated worldwide, in 2015, was 71.4 years—73.8 years for women and 69.1 years for men. The countries with higher values for life expectancy (over 83.0 years) were Japan, Switzerland, and Singapore. At the opposite end, with the lowest life expectancy at birth in 2015, was Sierra Leone with 50.1 years.

Life expectancy at EU-28 level in 2017 was 80.9 years. Although significant progress has been made regarding population health, there are still large disparities for life expectancy between the EU member countries. The highest values of life expectancy at birth were recorded in Spain (83.3 years) and Italy (83.2 years). Bulgaria, Latvia, and Lithuania were at the opposite end, with life expectancy at birth below 75 years.

Most studies regarding life expectancy analyze the strong link between life expectancy and standard of living [8,9,10]. A person with a good standard of living has the resources to take care of their health and, in case of illness, to treat themselves.

Other factors influencing life expectancy are war, famine, and infectious diseases. Given the development of technology and medicine, some diseases have diminished, whereas for many others there is treatment. The problem arises in cases of cardiovascular diseases, which represent the main cause of premature death in Europe [11].

Most deaths are caused by circulatory system diseases and cancer; cardiovascular diseases recently decreased in countries with high income but remain the leading cause of death worldwide.

Although it is known that circulatory system diseases, such as cardiovascular disease, influence life expectancy, there are not many studies regarding the link between the two. This article presents the results of research that aim to establish a link between cardiovascular disease and healthy aging in Europe.

Life expectancy and related factors analyses are of big interest among academics, policymakers, medical researchers, and others in order to direct the flow of funds in the most effective way possible to the population groups in most need. High life expectancy and low birth rate will lead to the aging of the population, having profound implications on the school age population, politics, healthcare, labor force, social protection, social security issues, and public finances.

In this context, the aim of this paper is to identify the factors that influence longevity in Europe. In order to address this objective, we organized the rest of the article as follows: Section 2 contains the literature review, Section 3 is dedicated to describing the data and methods used, Section 4 presents the results obtained, and the last section focuses on highlighting the main conclusions.

## 2. Literature Review

Life expectancy reflects premature death, which across the world is registered as large differences in health [12]. Life expectancy measures the health and well-being of a population, which are indicators of the health output [13]. The life expectancy of a nation reflects both social and economic conditions and the quality of the medical infrastructure [14,15]. The decrease or stagnation in life expectancy is a cause for concern, signaling a decrease in the population health profile, influenced by adverse socio-economic trends, deterioration in the provision or quality of medical services, or worsening behavioral factors [16].

For 160 years, life expectancy has steadily increased by one-quarter of a year, which is considered an extraordinary human achievement. Although experts studying the phenomenon of mortality have claimed that life expectancy has reached the maximum threshold, the evidence presented in the Policy Forum suggests that life expectancy has not reached this limit [17].

The trend recorded by people’s life expectancy over the past hundred years has been a steadily rising one [18]. The recorded deaths were caused by epidemics and pandemic infectious diseases, famine, and war. In the middle of the 19th century, as living conditions improved and progress regarding public health and medical interventions occurred, infectious diseases declined [8].

Since 1940, predicted mortality had a significant influence on changes in life expectancy, and a 1% increase in life expectancy led to a 1.7–2% increase in population. Instead, there is no evidence that significant increases in life expectancy generate an increase in income per capita, as life expectancy has a small influence on GDP [19].

Mortality is influenced by socio-economic factors; Tarkiainen et al. analyzed how life expectancy changed over 20 years in the Finnish general population. It has been shown that mortality stagnates among low-income people, and alcohol-related illnesses increase mortality. Income influenced mortality more than occupation or level of education, perhaps because income more clearly identifies an economic segment in a social hierarchy. The results identified that poor people experience more health problems, a higher mortality rate, and a lower life expectancy [10]. The link between life expectancy and income was studied by Marks [19], Preston [20], Black et al. [21], Rao [22], and Prentice et al. [23].

In developed countries, morbidity and mortality are associated with status, housing, occupational class, overcrowding, education, unemployment [24,25], and income [26,27,28]. Mortality is lower in countries with equal income distribution [29], and, in developed countries, it is not closely linked to average income [9].

The relationship between income and life expectancy is well established but remains insufficiently understood. In the United States, a survey was conducted in the period of 2001–2014, and the results showed that high income was associated with high longevity, and life expectancy differences between groups of people with different incomes have increased over time. However, the association between life expectancy and income varied substantially across areas; smaller differences were observed related to health behaviors and local area characteristics [30].

Life expectancy and healthy aging imply good physical and mental health, and these are associated with high well-being [31]. Welfare is associated with income, as increasing income implies increasing well-being [32,33,34]. Welfare is indirectly determined by education [35,36], because education improves social relationships and generates higher income, which increase well-being [37] but not significantly [38].

Welfare is reflected nationally by GDP, being used as a proxy measure of development, assuming that wealth increases well-being [39,40]. In countries with high GDP levels, citizens will have good health and a longer life expectancy, as high living standards lead to disease prevention and treatment [41], and better health implies an increase in GDP [42]. GDP has a significant influence on life expectancy for most countries [43,44].

Other studies have correlated life expectancy with mortality caused by cardiovascular disease. In India, mortality data reflect that cardiovascular diseases represent one-quarter of the total mortality. Demographic projections suggest a major increase in cardiovascular disease mortality as life expectancy increases and the population growth changes [45].

In Russia, there has been a spectacular increase in mortality, as for 4 years life expectancy has decreased by 5 years. Many factors influence this phenomenon, including economic and social instability, high tobacco and alcohol consumption, inadequate nutrition, depression, and deterioration of the health system. These results clearly demonstrate that the major decline regarding health and life expectancy is influenced by economic and social situations and health problems [46].

Crimmins and Saito [47] analyzed healthy aging in the USA by gender and level of education and concluded that there are large racial and educational differences regarding healthy life expectancy, with education influencing healthy life expectancy more than life expectancy. Educational differences regarding healthy life expectancy have increased over time due to increased differences in mortality and morbidity. There was a compression of morbidity among people with a high education level and an expansion of morbidity among people with a low education level. The link between life expectancy and education was also studied by de la Croix and Licandro [48], Boucekkine et al. [49], Zhang and Zhang [50], and Zhang et al. [51].

Longevity was associated with education and per capita income, with Lutz and Kebede [52] highlighting that although there was a correlation between life expectancy, income, and education, the relationship between longer life and education was more linear and constant. A more complete education represents a good predictor of longevity, because a stronger culture leads to healthier lifestyle choices, for example, in nutrition or disease prevention (including cardiovascular diseases). The correlation between wealth and longevity is perhaps caused by the fact that better education also gives access to more prestigious and better paid jobs and, therefore, to greater possibilities for medical treatment and better nutrition. Therefore, income does not represent the primary cause of healthy aging and longevity.

Regarding the differences regarding gender, Luy and Minagawa [53] focused on the proportion of life spent in poor health, and the analysis suggested that women’s longevity advantage translates into health disadvantages relative to men. The results indicated that women suffer from poor health not in spite of living longer, but because they live longer.

Yong and Saito [54] examined the increase in life expectancy of Japanese people by gender related to their health status from 1986 to 2004. Using the Sullivan prevalence method, the results showed that for both sexes and all ages, life expectancy before 1995 was higher in the years of well-evaluated health than subsequently in years of poor health. Until 1995 there was evidence of compression of morbidity, followed by an extension of morbidity.

In the Netherlands in the period of 1992–1997, healthy aging was studied regionally within the healthcare system. The results showed that healthy life expectancy presents a regional pattern, slightly different from life expectancy and self-reported health. The regional distribution of healthy life expectancy is different by gender, especially at the age of 65. The healthy life expectancy of women aged 65 is independent of overall life expectancy. Social conditions and lifestyle differences between regions are inversely associated with healthy life expectancy, while health care variables have no influence [55].

Jagger et al. [56] studied healthy aging in 25 EU countries and observed that although life expectancy in the European Union (EU) is increasing, it is not known if these additional years are spent in good health. The results showed that in 2005, a man aged 50 presents a life expectancy of 67 years, of which there are 3 years without restrictions of activity, and a woman presents a life expectancy of 68 years, from which there is 1 year without restrictions. Healthy life expectancy differs by gender, but also by country, and these differences are bigger than in the case of life expectancy between countries. Healthy life expectancy at 50 years was directly associated with GDP, learning, and expenses for the care of the elderly, and inversely associated with long-term unemployment for men.

Mathers et al. [57] produced the first estimates of healthy life expectancy for 191 countries in 1999, using the Sullivan method. These were based on estimates of the incidence, prevalence, and disability distribution for 109 causes of disease and injury by age group, sex, and region and an analysis of 60 representative health surveys around the world. The results showed that Japan had the highest average healthy life expectancy of 74—5 years at birth in 1999. In countries where the HIV-AIDS epidemic is the most widespread, the healthy life expectancy at birth is less than 35 years. Healthy years of life lost due to disability represent 18% of total life expectancy and fall to about 8% in countries with the highest healthy life expectancies. Globally, the difference between men and women is smaller for healthy life expectancy than for total life expectancy. Healthy life expectancy grows between countries faster than life expectancy, which suggests that reductions in mortality are accompanied by reduced disability. Women are living longer, but they spend more time with disabilities. The link between health care spending per capita and healthy life expectancy is much stronger than the link between health care spending per capita and life expectancy.

Healthy life expectancy is a global indicator of changes in population health. Robine and Ritchie [58] analyzed all the studies known in that period in the United States, continental Europe, Canada, and the United Kingdom that used Sullivan’s method of calculating life expectancy without disabilities. The results showed that the average healthy life expectancy was 60 years for men and 64 for women, with the proportion of years of disability ranging from 11 to 21% in men and from 14 to 24% in women. At age 65, men can expect 8 years of life without disabilities and women 10 years, with life expectancy being 14 and 19 years, respectively. The difference between the richest and the poorest countries was 6.3 years for life expectancy and 14.3 for healthy life expectancy in the case of men and, respectively, 2.8 and 7.6 years for women. These results suggested that differences in health are greater between social groups than between gender. Diseases that affected life expectancy the most were circulatory diseases, cancer, and accidents, and diseases that affected healthy life expectancy the most were circulatory diseases, locomotor disorders, and respiratory disorders.

Groenewegen et al. [55] analyzed life expectancy at birth and healthy life expectancy at age 65 for men and women in the Netherlands, highlighting the factors that influence regional variations. The study was conducted in 27 health care regions, and life expectancy and healthy life expectancy were calculated using the 1995 mortality data and the health interview survey data (1992–1997) from the Netherlands Statistics. Healthy life expectancy presents a regional pattern, slightly different from the pattern of life expectancy and self-reported health. The regional distribution of healthy life expectancy differs according to gender, especially at 65 years of age. The healthy life expectancy of women aged 65 is independent of their overall life expectancy. Social conditions and lifestyle differences between regions are inversely associated with healthy life expectancy in the Netherlands regions, and health care variables show no clear relationship.

In order to analyze life expectancy and healthy life expectancy of Chinese people, a study was conducted in the period of 1990–2015, both at the provincial and national level. The results showed that life expectancy was 4.4 years higher than the global averages, and the healthy life expectancy was 5.2 years higher, in the case of women presenting higher values than in the case of men. In developed areas, high values of life expectancy and healthy life expectancy were recorded, while low values were present in underdeveloped regions [53].

Lai et al. [59] studied the association between mortality and the burden of healthcare outside China and whether there are other confounding factors, such as the level of healthcare. The results showed that the rapid increase in cases in a short time can lead to more cases and even more deaths. Mortality was not associated with health care level, which can be explained by the fact that countries with more advanced health care systems have a better diagnostic capacity to identify more cases.

Life expectancy is positively correlated with the health care system, with differences regarding the quality of health care and medical staff contributing to a health gap [60]. Public health and medical care expenditures influence the health and quality of life of the population and, therefore, life expectancy [60,61].

Another variable associated with life expectancy is the individual’s satisfaction regarding life. Life satisfaction refers to the tendency to report aspects of life in general as good [31]. In Latin America, individuals reported significantly higher than average levels of life satisfaction. In Australia, 83% of Australians and Americans viewed all positive emotions as inherently desirable compared to only 9% of Chinese who shared the same opinion [62]. Thus, differences between cultural norms and values influence how people report feelings about their lives [63].

Acknowledging the factors influencing healthy aging, the following hypotheses were formalized in order to highlight the influence of cardiovascular diseases on healthy life expectancy:

**Hypothesis** **1** **(H1).** *Socio-economic factors that are correlated to longevity are also correlated to healthy aging;*

**Hypothesis** **2** **(H2).** *Cardiovascular diseases significantly influence healthy aging, and there are significant differences between genders;*

**Hypothesis** **3** **(H3).** *The countries clustered according to healthy aging and longevity are also clustered according to GDP.*

## 3. Research Methodology

In general, the main factors that influence healthy aging and longevity are genetic problems, in particular cardiovascular diseases, the main cause of premature death in Europe, and people’s income.

Income influences life expectancy in two ways: (a) directly: people with high income may benefit from certain medical and pharmaceutical facilities (medicines, complex analyses and investigations, healthy lifestyle, and specialized centers); and (b) indirectly: a person with a high standard of living can enjoy safe material comfort and also has a mentality of development, health, and well-being.

Thus, an analysis was performed considering life expectancy, healthy life expectancy, average income, cardiovascular diseases/1000 inhabitants, average satisfaction, and GDP/capita. Other analyses were performed considering healthy life expectancy for both men and women.

Life expectancy at birth is the average number of years that a newborn has to live if they live the rest of their life, in terms of age-related mortality over the reference period of the mortality table.

Healthy life expectancy is defined as the average number of years still to be lived by a person at 65 years of age, if they are subjected to the current mortality conditions for the rest of their life. This represents the average number of years lived without suffering from diseases and disabilities, enjoying full health.

The average income is measured on the purchasing power standard.

The number of cardiovascular disease represents the number of deaths caused by the circulatory system (most of them are cardiovascular disease). The variable rate of cardiovascular disease influences life expectancy and healthy life expectancy because most premature deaths in Europe and worldwide are caused by genetic transmission of cardiovascular diseases. Since the exact number of hereditary cardiovascular diseases is not known, in the analysis, the rate of cardiovascular disease that causes mortality early was used.

Average satisfaction refers to how satisfied people are with their life overall, for a person older than 16 years old.

GDP (gross domestic product, PPS) is reported per number of inhabitants as volume indices of real expenditure per capita.

Education represents the number of pupils and students with a bachelor’s or equivalent level.

All data for the last year for the European Union are available (2019, and in some cases, 2018).

Using the correlation analysis, we indicated the link between healthy aging and cardiovascular diseases. In order to highlight the factors influencing healthy aging, we performed the principal components analysis (PCA) using the following variables: income, cardiovascular diseases, life expectancy (both for men and women), healthy life expectancy (both for men and women), average satisfaction, education, and GDP/capita. After detecting the variables impacting healthy aging, we performed the regression analysis, considering healthy life expectancy as the dependent variable and cardiovascular diseases, GDP/capita, income, education, and average satisfaction as independent variables. Other regression analyses were performed according to gender, considering healthy life expectancy (both for men and women) and cardiovascular diseases, in order to evidence if there are differences according to healthy aging between men and women.

In order to classify countries by healthy aging and longevity, we performed the cluster analysis, comparing clustering according to GDP, highlighting whether countries are similarly classified according to GDP and healthy life expectancy and life expectancy.

The methods used were correlations, regression analysis, hierarchical cluster analysis, and principal component analysis.

A phenomenon is the result of the action of one or more factors. A linear regression model identifies the variables when the models used to estimate the model parameters are known [64]. The regression analysis implies the existence of a statistical link between the behavior of some variables [65]. The linear regression models, its variants, and extensions represent the most useful and used statistical tools for research [66].

Multivariate regression analysis model is formulated as follows:

(1) $$y=\beta_{0}+\beta_{1}X_{1}+\beta_{2}X_{2}+\ldots +\beta_{n}X_{n}+\varepsilon$$

The assumptions of multivariate regression analysis are normal distribution, homoscedasticity, autocorrelation, and non-existence of strong correlations between the independent variables.

Another method used to analyze heathy life expectancy is the analysis of the principal components analysis. The main principle of this method is to extract the smallest number of components to reflect the total information contained in the original data; the new components express new attributes of individuals, which are constructed so that they are not correlated with each other, and the new variables represent a linear combination of the original variables [67].

This method provides a graphical visualization of the individuals according to the similarities between them. The principal components analysis is defining the elements of the initial data table and the way of calculating the distances between points. This method can only be applied to quantitative variables and large tables that contain information on more than 15 individuals and 4 variables [68].

Its goal is to extract the important information, to represent it as a set of new orthogonal variables, and to display the pattern of similarity of the observations and of the variables as points in maps [69].

Principal component analysis (PCA) was used with the main purpose of synthesizing a smaller number of new variables as much as possible of the variation of the initial variables in order to obtain the fundamental factors that explain the differences between countries in terms of life expectancy. In this respect, the scores of the principal components obtained in this stage were used in the cluster analysis for grouping the countries.

The vector that explains the most variance of the data is the first eigenvector. Likewise, the second vector that explains most of the remaining variance is the second eigenvector.

Cluster analysis groups the elements of a sample so as to minimize the statistical variance between the elements of the group and to maximize the variance between groups [70]. For efficient use of the cluster analysis, it is essential to select the appropriate grouping algorithms [71].

There are four methods used for grouping: hierarchical algorithms, partitioning, overlapping, and ordering; the algorithms are validated through the method’s capability to regain the cluster structure [72]. The k-means methodology aims to ensure a consistent grouping, which is necessary for knowing the number of n_C_ clusters in which the data are classified. Data grouping is based on Euclidean distances [73].

Hierarchical algorithms construct a tree-like structure; the most well-known five agglomerative algorithms are the simple link, the complete link, the middle link, the center method, and the Ward method [74].

Hierarchical cluster is a method that organizes objects into a dendrogram, whose branches represent the desired clusters. The process of cluster detection is referred to as tree cutting, branch cutting, or branch pruning [75]. This method is an effective method for forming scales from sets of items [76].

Hierarchical cluster analysis creates a unique set of categories or clusters by sequentially associating variables, clusters, or variables and clusters. At each stage, starting with the correlation matrix, all untested groups and variables are tested in all possible pairs, and the pair presenting the highest average correlation in the test cluster is chosen as the new cluster. A graph, constructed as the taxonomic dendrogram of the biological systematist, shows the class inclusion relationships between clusters and the value of the clustering criterion associated with each one [77].

Hierarchical cluster analysis implies collection methods seeking to construct a hierarchically arranged sequence of partitions for some given object set. This method results in a hierarchy based on proximity measures defined for every pair of objects [78].

The analysis was conducted on 30 European countries for the years 2015, 2016, 2018, and 2019. The data source is the European Union databases—the European Center for Disease Prevention and Control Cases of COVID-19; the statistical and econometric analysis was performed with EViews, Excel Tableau, and SPSS software.

## 4. Research Results

In 2019, across European countries, life expectancy records a minimum of 75.1 years and a maximum of 84 years. These values show that in Europe, life expectancy is medium and high. The mean is 80.69 years and the standard deviation is 2.79, emphasizing that the countries of Europe are homogeneous regarding life expectancy. The countries from western Europe present higher values regarding life expectancy. These countries are developed countries, highlighting a connection between life expectancy and the standard of living and the degree of development of the country.

Regarding healthy life expectancy, the minimum value is 53.1 years, the maximum is 73.3 years, the mean is 62.59 years, and the standard deviation is 5.32.

**Figure 1:**
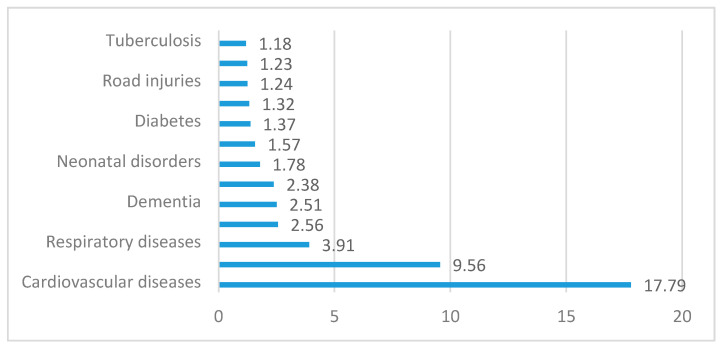
Number of deaths globally (million).

According to data, most deaths are caused by circulatory system diseases and respiratory diseases globally (Figure 1). Circulatory system diseases and cancer represented 62% of all death causes in 2016, while in 1900 they represented only 18%. The circulatory system has an important role for the biological human, being formed by a heart, blood, and lymphatic vessels that make up a functional unit coordinated and permanently adapted to the needs of the organism.

Cardiovascular diseases remain the leading cause of death worldwide, although over the past two decades, cardiovascular mortality has declined in countries with high income. At the same time, cardiovascular deaths and cardiovascular diseases have presented an increased trend in countries with low and middle income. The share of deaths due to cardiovascular diseases represents 4% of total deaths in countries with high income and 42% in countries with low income. The large difference regarding the number of deaths caused by cardiovascular diseases highlights that it is significantly influenced by the conditions and standard of living.

From all diseases of the circulatory system, the main causes of mortality among the elderly are ischemic heart disease and cerebral vascular diseases. Others diseases of the circulatory system include those related to high blood pressure, cholesterol, diabetes, and smoking, which are strongly associated with stress.

Therefore, the link between cardiovascular diseases and healthy life expectancy was analyzed.

**Figure 2:**
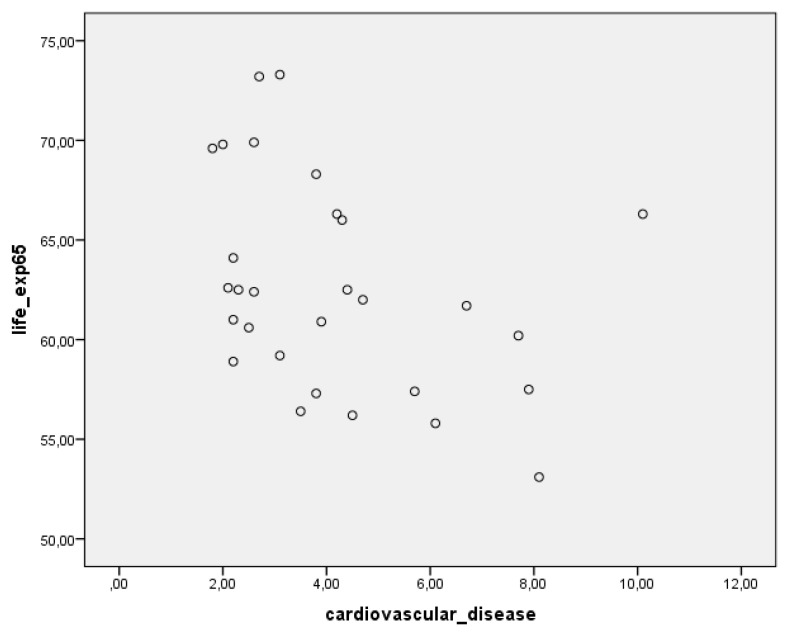
Correlogram of healthy life expectancy and cardiovascular diseases/1000 inhabitants, EU, 2019.

As we can see from the correlogram (Figure 2), the link between life expectancy and cardiovascular diseases across the European Union is inverse; thus, as the number of deaths caused by cardiovascular diseases increases, healthy aging decreases.

**Figure 3:**
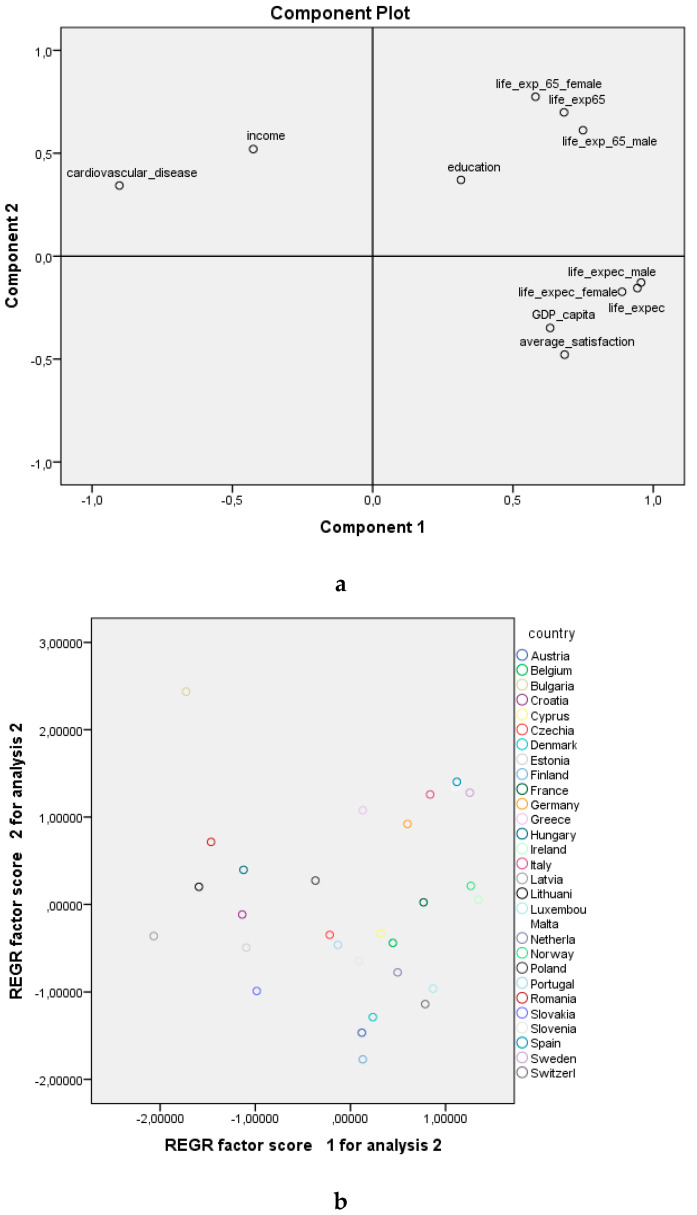
Graphical representation of variables (**a**) and countries (**b**) in the axis system. REGR—regression.

Using the principal components analysis (Figure 3a), referring to the first two factorial axes we can affirm that between the GDP/capita, average satisfaction, and life expectancy the link is direct, with these variables located on the first factorial axis. Between education (bachelor’s degree), healthy life expectancy, and education the link is direct, confirming that education contributes to healthy aging. Although the factors that influence longevity are different from those that influence healthy aging, all of these factors are inversely correlated with the number of cardiovascular diseases and income. This analysis does not confirm Hypothesis 1, according to which the factors that are correlated to longevity are also correlated to healthy aging, but confirms the fact that the number of cardiovascular diseases reduces longevity and healthy aging.

Overlapping the two charts (Figure 3a,b), the countries in the EU registering high values for the number of deaths caused by cardiovascular diseases are Bulgaria, Lithuania, Hungary, and Serbia. Life expectancy is higher in Luxembourg, Italy, Switzerland, Iceland, Norway, Malta, Sweden, Cyprus, and France.

In order to find a relationship between healthy aging and socio-economic variables, a linear regression model was generated for the EU, 2019. According to Table 1, only the variable of cardiovascular diseases significantly influences healthy life expectancy, with a probability of 90%.

The parameter estimates are as follows: if the number of deaths caused by cardiovascular disease (numerical variable) increases with one unit, healthy life expectancy decreases on average by 1.307 years (indirect relationship), holding all the other variables constant, considering a probability of 90%.

According to gender, for the EU, 2019, considering a probability of 95%, cardiovascular diseases significantly influence healthy aging in the case of males but not in the case of females (Table 2), confirming Hypothesis 2, according to which cardiovascular disease significantly influences healthy aging and there are significant differences between genders. The main causes of death among men are heart disease, cancer, trauma, chronic respiratory disease, and stroke. There are multiple reasons in the literature explaining the differences between genders considering mortality, the larger figure for men is explained by the male lifestyle. According to experts from Harvard Medical School, men take higher risks, have more dangerous jobs, are more prone to heart disease at a younger age due to lower estrogen, are more likely to commit suicide than women, are less socially connected, and avoid going to the doctor. The share of men that consume alcohol weekly in the EU is higher than the share of women (38% of men aged 18 and over compared to 23% of women in 2014). In total, 24% of men aged 18 years and over smoke daily compared to 16% of women. Unlike smoking and alcohol consumption, regular consumption of fruits and vegetables is considered an important element of a healthy and balanced diet. In the EU in 2014, 49% of men ate between one and four servings of fruits and vegetables daily compared to 54% of women. Smoking, alcohol, and eating healthy have an impact on weight. In the EU in 2014, 57% of men were considered overweight compared with 44% of women. The standard retirement age is 65 years for men and 61 years for women. Since 1990, women have benefitted for a period of time raising children (2 years, and 3 years in cases where the child has problems). The data indicate that women in Romania use medical services more often than men do: 82.6% of men never went to the doctor, whereas 72.9% of women did.

Using the hierarchical cluster analysis, we classified the countries into four different clusters:

- First cluster includes Iceland, Sweden, Norway, Finland, France, Italy, Spain, Malta, and Cyprus;
- Second cluster includes Germany, Ireland, Denmark, Belgium, Portugal, Greece, Slovenia, and Austria;
- Third cluster includes Turkey, Czechia, Croatia, Slovakia, Hungary, and Estonia;
- Fourth cluster includes Romania, Bulgaria, Serbia, and Latvia.

**Figure 4:**
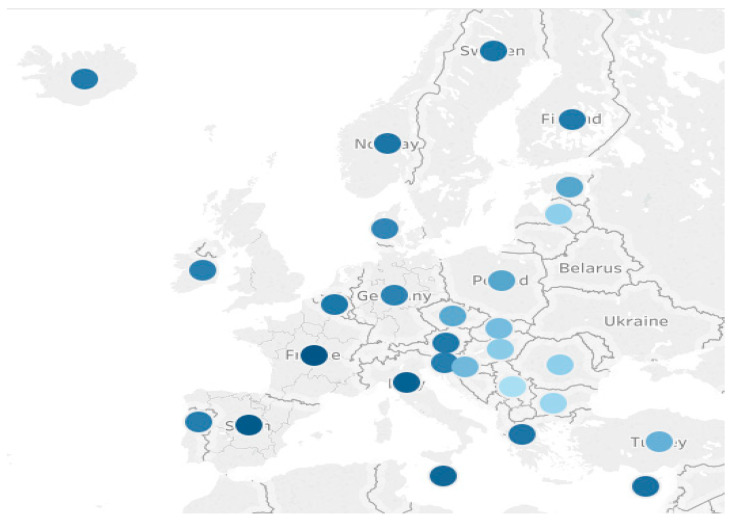
Clustering regarding life expectancy and healthy life expectancy.

It can be seen (Figure 4) that regarding life expectancy and healthy life expectancy, the countries in the EU grouped differently according to GDP, invalidating Hypothesis 3, according to which the countries clustered according to healthy aging and longevity are also clustered according to GDP. This result is also supported by the result of the regression analysis, according to which a probability of 95% GDP/capita does not significantly influence healthy aging. Although there is a positive link between healthy aging and GDP/capita (reflecting the standard of living), the link is not so strong at EU level.

## 5. Conclusions

Lately, in Europe, cardiovascular diseases are the strongest cause of mortality, especially cardiovascular diseases transmitted genetically. Another issue is that developed countries, characterized by high income and better technologies and treatments, will experience a higher life expectancy.

The analysis involved variables for the year 2019: healthy life expectancy, life expectancy, income, education, GDP, and cardiovascular diseases for the European countries. The conclusion of the study is that for the EU, healthy aging is influenced by cardiovascular diseases, with significant differences existing between men and women.

By analyzing life expectancy and healthy life expectancy, a growing trend is observed; life expectancy has increased more than healthy life expectancy, in other words, people are living longer but not so healthy. The variables of average satisfaction, education, income, and GDP do not significantly influence healthy life expectancy, although they are correlated with it. These variables have an influence on life expectancy up to 65 years of age, and after this age, life expectancy is influenced by life expectancy at birth and lifestyle until this age; the influence of other variables is small or insignificant.

Healthy life expectancy for women is higher than for men, and this is valid all over the world, regardless of living conditions, women’s status, or other factors. The explanations for this difference are not entirely reliable. Traditional arguments are based on social and environmental factors: throughout history, men have consumed more tobacco, alcohol, and drugs than women in most societies and are more likely to die from associated conditions, such as lung cancer, tuberculosis, and cirrhosis. The biological differences appear because women have a higher resistance to infections and degenerative diseases.

By clustering the countries according to healthy aging, we obtained four different clusters that also differed according to GDP.

Life expectancy and related factors analyses are of big interest among academics, policymakers, medical researchers, and others in order to direct the flow of funds in the most effective way possible to the population groups in most need.

Further research should aim to include other variables and chronological components. In addition, depending on the possibilities of accessing the specific data series, the study can also be applied all over the world.

## Figures and Tables

**Table 1:** Regression analysis.

Variables	B	Std. Error	t	Sig.
Constant	72.82	19.409	3.752	0.001
Cardiovascular diseases	−1.307	0.748	−1.747	0.094
GDP/capita	0.13	0.031	0.438	0.666
Income	0.303	0.316	0.961	0.347
Average satisfaction with life	−1.686	2.368	−0.712	0.483
Education	2.99 × 10^−6^	0.000	1.43	0.166

**Table 2:** Regression analysis male/female.

	Model I (Male)				Model II (Female)			
Variables	B	Std. Error	t	Sig.	B	Std. Error	t	Sig.
Constant	67.142	2.015	33.318	0.000	65.413	2.131	30.690	0.000
Cardiovascular diseases	−1.164	0.430	−2.705	0.012	−0.606	0.455	−1.331	0.194
